# Organisational contextual drivers of evidence-based practice across acute and primary care

**DOI:** 10.1186/s12912-025-04129-y

**Published:** 2025-11-22

**Authors:** Jude Ominyi, Aaron Nwedu, Ukpai Eze, Anastasia Ngon, Uchenna Chima

**Affiliations:** 1 School of Health, Sciences & Society, University of Suffolk, Ipswich, UK; 2 David Umahi Federal University of Health Sciences, Uburu, Ebonyi State Nigeria; 3 Faculty of Health, Medicine and Society, University of Chester, Chester, UK; 4 Faculty of Business & Law, University of Northampton, Northampton, UK; 5 School of Nursing and Allied Health, Birmingham Newman University, Birmingham, UK

**Keywords:** Evidence-based practice, Organisational context, Leadership, Social capital, Nursing workforce, Implementation, Primary care, Acute care, Professional development

## Abstract

**Background:**

Evidence based practice (EBP) is widely recognised as fundamental to high quality nursing care, yet implementation remains uneven across healthcare settings in England. Attention has shifted from individual barriers to organisational context. Leadership, team dynamics, access to resources, and social capital shape how nurses engage with EBP. Despite national policies promoting research active environments, how these ambitions are realised at the frontline is unclear. This study examined how organisational factors influence nurses’ implementation of evidence across acute and primary care.

**Methods:**

A cross-sectional design was used with registered nurses working in acute and primary care settings. Two validated instruments, the Evidence Based Practice Implementation Scale and the Alberta Context Tool, were administered. A nonprobability sampling strategy targeted the acute and general practice nursing workforce. Response distributions were monitored across pre specified strata and fieldwork closed once coverage and precision criteria were met. Descriptive statistics summarised participant and organisational characteristics. Inferential analyses compared settings, mediation modelling tested the role of social capital in the leadership to EBP pathway, and cluster analysis identified implementation profiles.

**Results:**

Engagement with EBP was moderate overall (M = 3.16, SD = 0.88) with no significant difference between sectors (*p* = 0.38). Acute care nurses reported higher leadership support (M = 4.01 versus 3.78, *p* = 0.008) and better access to structural resources (M = 3.35 vs. 3.10, *p* = 0.004). Within acute care, leadership differed across specialties, with higher scores in ICU or CCU and general medicine, F (4, 636) = 4.12, *p* = 0.003. Social capital significantly mediated the association between leadership and EBP implementation (*β* = 0.15, 95% CI 0.10–0.21). Three engagement clusters were identified, high 32%, moderate 45%, and low 23%, each with distinct organisational profiles.

**Conclusion:**

Organisational context, particularly leadership and social capital, is central to nurses’ capacity to implement evidence. Variation across specialties and sectors indicates that a one size fits all approach is unlikely to succeed. Policy relevant levers include formalising protected time, resourcing embedded facilitation, investing in knowledge infrastructure, and expanding clinical academic pathways, to create environments where evidence use is routine and supported.

**Clinical trial number:**

Not applicable.

## Background

Evidence-based practice (EBP) remains central to the delivery of high-quality healthcare, supporting nurses to integrate their clinical expertise with the best available research and the individual preferences of those receiving care [1, 2]. Across England, the implementation of evidence-informed nursing is increasingly embedded in policy and professional frameworks. Regulatory and advisory bodies such as the Nursing and Midwifery Council (NMC) and the National Institute for Health and Care Excellence (NICE) continue to emphasise the value of systematic evidence use in clinical decision-making [3, 4]. Despite this policy support, wide variation remains in how EBP is applied within practice. Earlier studies have linked this variation to individual-level factors including limited research literacy, time pressures, and lack of formal training [5, 6]. These concerns are valid, but an individualised lens overlooks the broader organisational context that plays a decisive role in shaping how, and to what extent, nurses adopt research-based approaches [7, 8].

Organisational context refers to the interrelated structural, cultural, and relational elements that define a work environment [7]. These include leadership behaviours, team norms, workplace culture, access to evaluation systems, and availability of clinical and technological resources [7, 9]. Across diverse settings, leadership has consistently been shown to influence engagement with research-informed practice [8, 10]. Managers and senior nurses can enable evidence use through behaviours that model curiosity, support learning, and prioritise reflection within teams [11, 12]. Organisational culture, defined through shared assumptions and routines, has a similarly powerful influence [13, 14], when teams collectively value innovation and critical enquiry, research-based care becomes more feasible and more aligned with day-to-day clinical work [14].

Recent literature has highlighted the critical role of organisational context in shaping whether and how nurses engage with research-informed care [12, 13]. Studies focusing on England have drawn attention to the relational and structural dimensions of this process, with particular emphasis on leadership, social networks, and infrastructure [11]. Similarly, a qualitative study by Ominyi & Alabi [9] reports that leadership behaviours were strongly associated with nurses’ confidence in using evidence, especially when team culture supported experimentation and learning. Besides, organisational slack and protected time were identified as significant enablers of EBP, but only when paired with strong peer support and managerial encouragement [12]. These findings suggest that organisational features do not operate in isolation but work together to shape nurses’ capacities and motivations.

The concept of social capital has further advanced this conversation by pointing to the importance of peer relationships and informal learning structures 14]. Interactions with colleagues, mentoring networks, and interdisciplinary exchanges all influence the degree to which research knowledge is shared and applied [14, 15]. These forms of relational support often fill gaps left by more formal mechanisms, especially in resource-constrained settings [15]. Evidence from recent evaluations of practice-based networks in England supports the idea that informal team dynamics can significantly improve implementation outcomes, particularly when they supplement limited access to formal training or resources [16].

Policy agendas in England, such as the NHS People Plan, have called for stronger alignment between workforce development and research activity [17]. These ambitions are reflected in frameworks that advocate for research-active teams and organisation-wide capability building [17, 18]. However, empirical evidence on how such ambitions translate into frontline nursing remains patchy. This study addresses that gap through a national survey of nurses working in both acute and primary care settings in England. Rather than comparing acute and primary care as discrete systems, the study considers how conditions across both sectors combine to influence practice. Findings are intended to contribute to ongoing debates around organisational development, workforce capacity, and leadership for evidence-based care.

### Aims

This study investigated how organisational context influence the implementation of EBP across healthcare settings in England. The specific objectives were to:

1. Assess the influence of leadership behaviours on the development of research-engaged clinical environments.
2. Examine how organisational culture affects the perceived relevance and everyday feasibility of evidence use in nursing practice.
3. Explore how social capital, including peer relationships, informal networks, and mentorship, facilitates or constrains EBP engagement.
4. Identify structural disparities in resource access and organisational support that affect the capacity for EBP across acute and primary care settings.
5. Investigate how leadership and social capital interact to shape EBP implementation, using mediation modelling to assess relational pathways.

## Methods

### Research design

This study employed a cross-sectional design, selected for its suitability in capturing data from a large and geographically dispersed nursing workforce during a defined time [19]. The approach enabled collection of rich organisational and behavioural data without imposing extended demands on participants, which is important given operational pressures in clinical practice [20, 21]. Cross sectional designs using online questionnaires offer both breadth and efficiency where staff availability is limited and research participation competes with clinical priorities [19, 22].

### Measures

This study employed two previously validated instruments and did not involve the development of new measurement items. The Evidence-Based Practice Implementation Scale (EBPIS) [23] was used to assess the frequency with which nurses engaged in evidence-based behaviours, including generating clinical questions, appraising and applying research, and supporting colleagues in evidence use. The EBPIS has demonstrated high internal consistency in diverse clinical settings, with Cronbach’s alpha values consistently reported between 0.92 and 0.95 [24, 25]. The behavioural frequency version of the EBPIS was used, with responses rated from 1 (never) to 5 (very often), in order to preserve fidelity to the validated implementation format rather than attitudinal adaptations that use agreement-based anchors.

Organisational context was measured using the Alberta Context Tool (ACT) [26], which captures key contextual domains relevant to evidence implementation, including leadership, workplace culture, evaluation processes, social capital, and access to structural and electronic resources. These domains align directly with the study objectives and reflect core dimensions of context identified in implementation science. For instance, leadership and culture relate to Objectives 1 and 2, evaluation systems align with Objective 3, social capital reflects Objective 4, and access to organisational resources supports Objective 5. The ACT has demonstrated robust psychometric performance internationally, with Cronbach’s alpha values across domains ranging from 0.54 to 0.91 [27–29]. Items were scored on a five-point scale from 1 (strongly disagree) to 5 (strongly agree). Table 1 presents the structure and internal reliability values for all ACT domains.

**Table 1:** Structure and reliability of the ACT

Scales	Items and Range	Cronbach’s Alpha
Leadership	6 items, 6–30	0.91
Culture	6 items, 6–30	0.86
Evaluation	6 items, 6–30	0.91
Social capital	6 items, 6–30	0.77
Formal interactions	4 items, 4–20	0.60
Informal interactions (non-direct care)	4 items, 4–20	0.75
Informal interactions (direct care)	5 items, 5–25	0.70
Structural and electronic resources (formal)	4 items, 4–20	0.71
Structural and electronic resources (traditional)	3 items, 3–15	0.60
Structural and electronic resources (electronic)	3 items, 3–15	0.54
Organisational slack (time)	4 items, 4–20	0.74
Organisational slack (space)	3 items, 3–15	0.63
Organisational slack (human resources)	2 items, 2–10	0.83

### Participants and recruitment

Participants were registered nurses employed in clinical roles across acute and primary care settings. Eligible nurses had been in their current post for at least six months to ensure familiarity with the organisational context. Nurse managers were included on this basis, given that many combine managerial and clinical responsibilities, particularly in senior ward-based or community facing positions.

#### Target population and denominators

Workforce data for 2022–2023 indicate that approximately 330,000 nurses were employed in acute care and around 22,000 were employed in primary care [30]. These figures provided the appropriate denominators for interpreting the coverage of our achieved sample of 1,001 nurses and are used in preference to the total number of nurses on the NMC register, which includes practitioners working outside the study’s scope.

#### Sampling aim and coverage

Sampling utilised a non-probability approach designed to maximise coverage across pre-specified strata relevant to EBP implementation, rather than to achieve statistical representativeness. Recruitment was undertaken through institutional mailing lists, professional networks, national nursing organisations, and relevant social media platforms, including X and LinkedIn, a common strategy for reaching dispersed clinical workforces [23]. Response distributions were monitored throughout fieldwork with respect to sector, English region, Agenda for Change band, years of experience, and, within acute care, specialty group.

#### Operationalising diversity and stopping rule

Diversity was defined a priori as achieving adequate coverage across the above strata to support planned comparisons. Fieldwork closed when two criteria were met. First, all strata were represented with sufficient numbers to enable between-group analyses as outlined in section “Data analysis”. Second, the achieved sample size provided acceptable precision for continuous outcomes given the observed variability, such that the 95% confidence interval around the EBPIS mean was approximately ± 0.06. These criteria ensured that diversity was achieved in a manner consistent with the study’s analytic aims while avoiding claims of statistical representativeness.

#### Missing data and denominators

Percentages are calculated on non-missing denominators. In Table 2, a Missing/Not stated row is presented for ‘work setting’ so totals reconcile to *N* = 1,001; for other variables, percentages are based on available cases.

### Data collection procedure

Data were collected between October 2022 and January 2023 using a secure online survey platform. The survey was accessible via mobile and desktop devices to facilitate flexible participation across shifts and work patterns. A total of 1,001 nurses completed the questionnaire, comprising 641 from acute care and 360 from primary care. Participants completed both the EBPIS and ACT instruments.

### Data analysis

Descriptive and inferential analyses were undertaken, guided by the study’s aims. All analyses were conducted using SPSS version 28 and R version 4.2.2. Descriptive statistics [31] summarised participant characteristics, including age, gender, qualifications, professional roles, years of experience, and current work setting (Table 2). Continuous variables were reported as means and standard deviations, while categorical variables were expressed as frequencies and percentages. Given the group sizes achieved (acute *n* = 641; primary *n* = 360), the study was adequately powered to detect small differences in ACT domain means (Cohen’s d ≈ 0.18–0.20), consistent with the focus on modest but policy relevant organisational effects [32, 33].

Independent samples t-tests and chi-square tests examined sectoral differences. Subgroup analyses within acute care compared five specialties (general medicine, surgery, ICU/CCU, emergency care, and specialist units). Mediation analysis tested whether social capital mediated the relationship between leadership support and EBP implementation, using structural equation modelling with 5,000 bootstrap resamples. Cluster analysis grouped participants into high, moderate, and low engagement profiles based on EBPIS scores. All tests were two-tailed with *p* < 0.05, and effect sizes were calculated and interpreted alongside significance tests [34].

### Ethical considerations

The study was conducted in accordance with the Declaration of Helsinki, which outlines ethical principles for medical research involving human subjects. Ethical approval for this study was obtained from the University of Bedfordshire Research Ethics Committee, Institute of Health, and Wellbeing (Reference: #2022/00204). The study did not involve NHS patients or clinical trials, and therefore approval from the Health Research Authority was not required in accordance with UK National Research Ethics Guidelines. All participants received an online participant information sheet that explained the purpose of the study, the voluntary nature of participation, data handling procedures, and their right to withdraw before submitting the survey. Electronic informed consent was obtained from every participant before they could access the questionnaire. Only participants who provided consent were able to proceed.

## Results

### Participant characteristics

As shown in Table 2, the sample reflected a wide distribution in terms of age, gender, educational background, and years of experience. These characteristics provide an important foundation for understanding how organisational context shapes engagement with EBP. The distribution was broadly similar across settings. Gender and age profiles were closely matched, with most nurses aged 35–50 years. Educational attainment was also comparable, though a slightly higher proportion of primary care nurses held master’s degrees. Most participants worked in publicly funded settings, although private sector representation was more evident in primary care. Just over one third of participants in both settings had more than 20 years of experience, suggesting a mature and professionally embedded workforce.

**Table 2:** Characteristics of participants

Variable	Acute (*n* = 641)	Primary (*n* = 360)	*p*-value
**Gender**			
Female	545 (85.0%)	305 (84.7%)	0.743
Male	90 (14.0%)	50 (13.9%)	0.812
Other	6 (1.0%)	5 (1.4%)	0.621
**Highest Education**			
Diploma	210 (32.8%)	110 (30.6%)	0.216
BSc	295 (46.0%)	165 (45.8%)	0.305
MSc	105 (16.4%)	75 (20.8%)	0.482
PhD/DProf	31 (4.8%)	10 (2.8%)	0.621
**Work Setting**			
Public	580 (90.5%)	300 (83.3%)	0.285
Private	50 (7.8%)	50 (13.9%)	0.412
Missing/Not stated	11 (1.7%)	10 (2.8%)	—
**Age**			
Mean (SD)	44.8 (10.4)	46.1 (10.9)	0.437
Under 35 years	135 (21.1%)	75 (20.8%)	0.512
35–50 years	256 (39.9%)	144 (40.0%)	0.689
Over 50 years	250 (39.0%)	141 (39.2%)	0.732
**Years of Experience**			
Mean (SD)	17.6 (9.8)	19.0 (10.8)	0.327
Under 10 years	170 (26.5%)	81 (22.5%)	0.501
10–20 years	255 (39.8%)	145 (40.3%)	0.614
Over 20 years	216 (33.7%)	134 (37.2%)	0.725

Note: “Missing/Not stated” row included in ‘work setting’ so totals reconcile to *N* = 1,001. Percentages are calculated on non-missing responses

### Organisational influences on EBP implementation

Mean scores from the EBPIS indicated moderate engagement with evidence-based behaviours across the sample (M = 3.16, SD = 0.88). No statistically significant difference was observed between nurses in acute care (M = 3.14, SD = 0.87) and those in primary care (M = 3.19, SD = 0.91), t (999) = 0.88, *p* = 0.38. These findings suggest that both groups engaged with activities such as appraising research, applying evidence, and supporting colleagues in similar ways. This directly relates to Objective 1, indicating that leadership practices alone may not fully explain implementation patterns. Differences across care settings in ACT domains such as leadership, culture, and access to resources are summarised in Table 3.

**Table 3:** Organisational context scores by care setting

ACT Domain	Acute (*n* = 641)	Primary (*n* = 360)	*p*-value
Leadership Support	4.01 (0.82)	3.78 (0.89)	**0.008**
Organisational Culture	3.91 (0.74)	3.83 (0.78)	0.062
Evaluation Mechanisms	3.76 (0.81)	3.68 (0.84)	0.073
Social Capital	3.84 (0.69)	3.61 (0.72)	**0.015**
Access to Structural Resources	3.35 (0.77)	3.10 (0.74)	**0.004**

Mean ACT domain scores for nurses working in acute and primary care settings. Bold p-values indicate statistically significant differences (*p* < 0.05)

Leadership support was stronger in acute care (M = 4.01, SD = 0.82) than in primary care (M = 3.78, SD = 0.89), reaching statistical significance (*p* = 0.003). *Although statistically significant, the difference in means was modest, and its practical significance should be interpreted with caution.* Differences in workplace culture (*p* = 0.067), evaluation systems (*p* = 0.071), and access to structural and electronic resources (*p* = 0.121) did not reach significance, although effect size analysis suggests they may still be practically relevant. These patterns align with Objective 2 and Objective 3, showing that while cultural and evaluative structures were present, they may not be robust enough to drive consistent engagement across settings. Organisational slack, particularly in relation to time and space, scored consistently low across both groups. This highlights a shared challenge across settings and addresses Objective 5 by pointing to structural conditions that limit research engagement. These results reflect behavioural frequency responses (1 = Never to 5 = Very Often) on the EBPIS.

### Variability within acute care settings

Subgroup analysis within acute care settings revealed more granular differences. Table 4*presents variation within acute care, highlighting how* leadership, culture, and evaluation support differ across general medicine, ICU/CCU, surgery, emergency, and specialist units. Leadership support varied significantly F (4, 636) = 4.12, *p* = 0.003, as did workplace culture F (4, 636) = 3.85, *p* = 0.004 and evaluation mechanisms F (4, 636) = 5.31, *p* < 0.001. General medicine and ICU/CCU reported the highest levels of support, while emergency and surgical nurses consistently scored lower. These differences further confirm that even within a single sector, organisational support is unevenly distributed. This reinforces Objective 1 and Objective 2 by showing that leadership and culture are not monolithic across service lines. Social capital and resource access showed less variation (*p* = 0.061 and *p* = 0.072, respectively), suggesting these elements may be more resilient or consistent across acute specialties. However, the near-significant results warrant attention, particularly for policy and practice development.

**Table 4:** ACT domain scores across acute care specialties

ACT Domain	General Medicine (*n* = 160)	Surgery (*n* = 120)	ICU/CCU (*n* = 100)	Emergency (*n* = 130)	Specialist Units (*n* = 131)	F (df)	*p*-value
Leadership	4.20 (0.55)	3.89 (0.66)	4.30 (0.52)	3.75 (0.68)	3.92 (0.75)	4.12	0.003
Culture	4.10 (0.63)	3.80 (0.59)	4.05 (0.65)	3.74 (0.60)	3.91 (0.72)	3.85	0.004
Evaluation	4.00 (0.70)	3.78 (0.60)	4.15 (0.58)	3.72 (0.55)	3.88 (0.61)	5.31	< 0.001
Resources	3.60 (0.55)	3.55 (0.60)	3.70 (0.52)	3.49 (0.57)	3.58 (0.63)	1.85	0.103
Social Capital	3.92 (0.59)	3.85 (0.64)	3.95 (0.62)	3.68 (0.70)	3.80 (0.77)	2.02	0.085

One-way ANOVA comparing ACT domain scores across five acute care specialties. Significant variation is observed in leadership, culture, and evaluation. Bold p-values indicate statistically significant differences (*p* < 0.05)

We did not conduct subgroup analyses across primary care roles or settings, as these environments typically lack the structured specialty divisions seen in acute care. The organisational profiles within primary care appeared more consistent, and there was no a priori justification or post hoc indication of meaningful intra-setting variability.

### Mediation effects of social capital

**Fig. 1:**
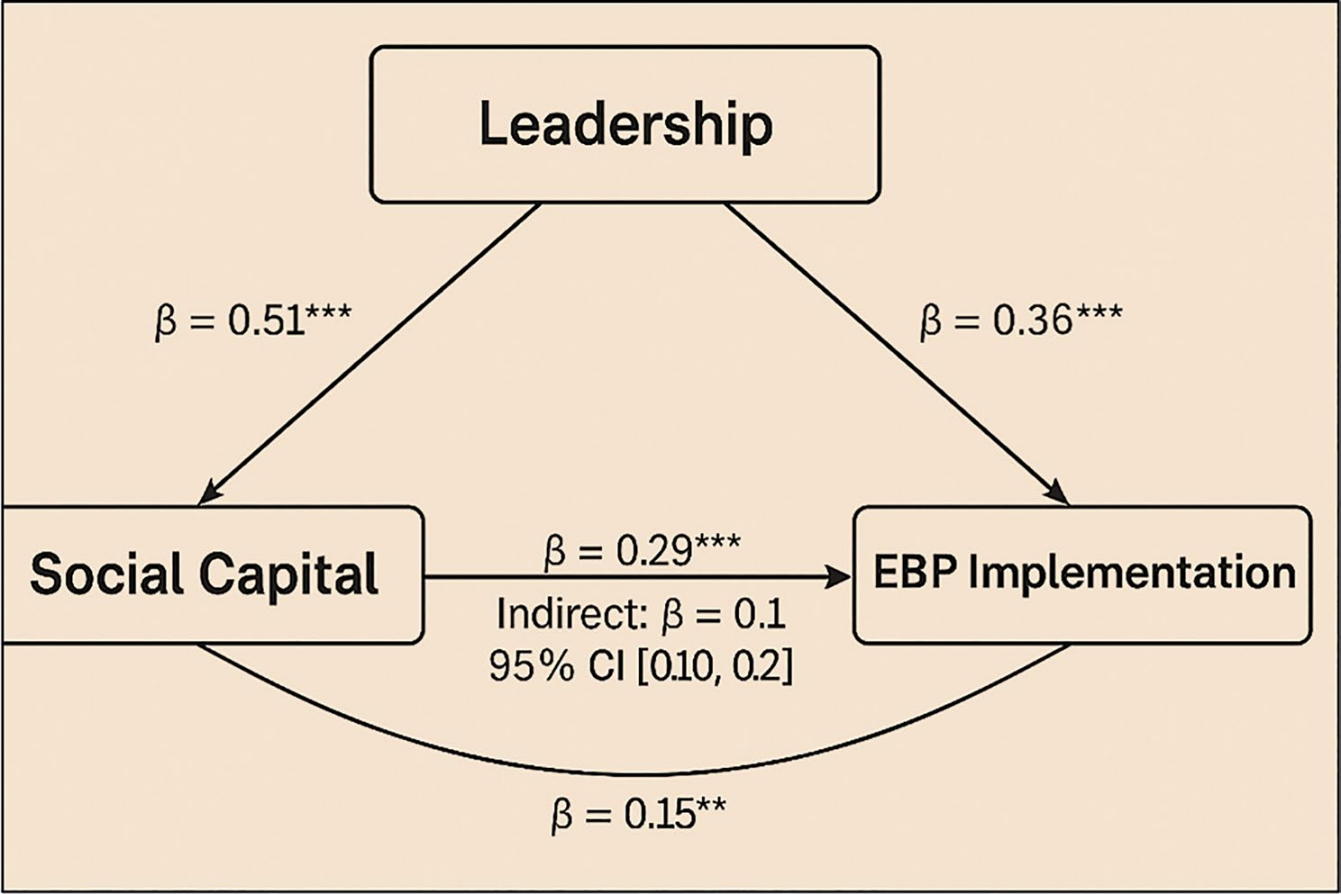
Standardised path coefficients for mediation model. Direct and indirect relationships between leadership, social capital, and EBP implementation. All path coefficients are standardised (β), and significance is set at *p* < 0.05

**Fig. 2:**
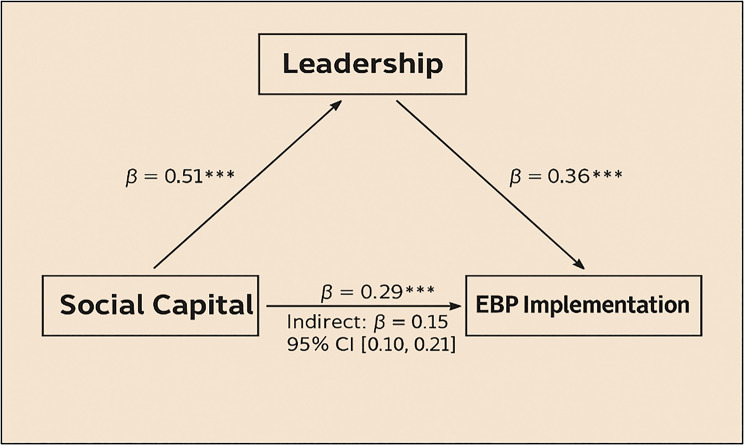
Mediation model linking leadership, social capital, and EBP implementation. Structural equation model showing relational pathways from leadership to EBP engagement, mediated by social capital. Indirect effects significant at 95% CI (0.10, 0.21)

Structural equation modelling examined whether social capital mediated the relationship between leadership and EBP implementation. The model demonstrated good fit indices, indicating that the proposed relationships between leadership, social capital, and EBP implementation were well supported by the data χ² (2) = 4.15, *p* = 0.13; RMSEA = 0.03; CFI = 0.99). Leadership support was positively associated with both social capital (*β* = 0.51, *p* < 0.001) and EBP implementation (*β* = 0.36, *p* < 0.001). Social capital also independently predicted EBP engagement (*β* = 0.29, *p* < 0.001). The indirect effect of leadership through social capital was significant (*β* = 0.15, 95% CI [0.10, 0.21]). These results provide clear evidence in support of Objective 4. Strengthening peer relationships and team cohesion may offer an effective mechanism for enhancing implementation in ways that are complementary to formal leadership structures. Standardised path coefficients from the mediation model are summarised in Fig. 1, while the full structural diagram is shown in Fig. 2 to illustrate the relational pathways between leadership, social capital, and EBP engagement.

### EBP engagement profiles

**Fig. 3:**
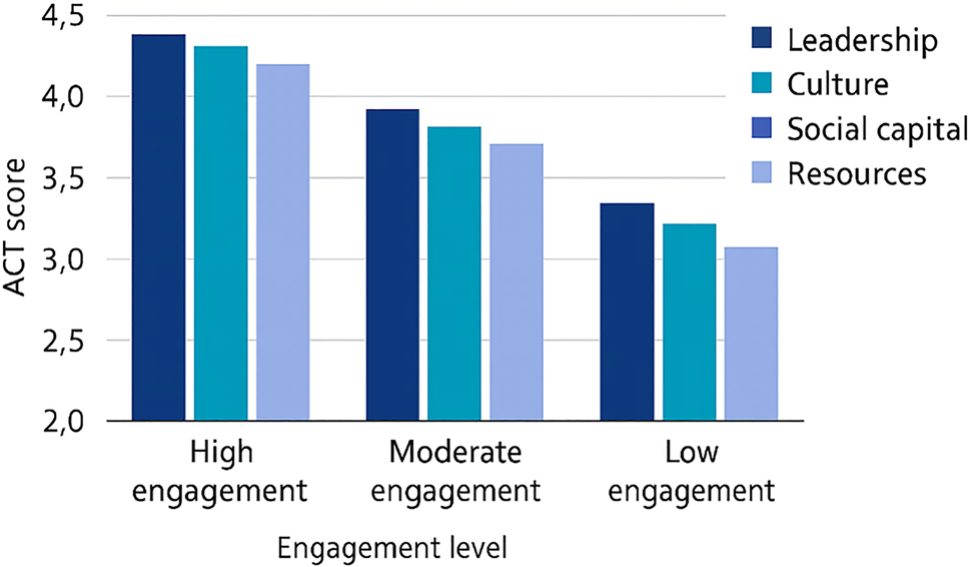
Cluster profiles and associated organisational scores. Differences in ACT domain scores across three nurse engagement groups (high, moderate, low). Higher scores reflect stronger organisational support for EBP

Cluster analysis identified three distinct groups based on EBP engagement: high (32%), moderate (45%), and low (23%). *A k-means clustering approach was used to group participants based on their EBPIS scores.* Differences in ACT scores across engagement clusters are visualised in Fig. 3, highlighting how high, moderate, and low engagement groups experience varying levels of leadership, culture, social capital, and access to resources. High-engagement nurses were present in both acute and primary care settings, but most commonly found in general medicine and high-functioning primary care environments. These areas reported strong leadership and workplace culture. In contrast, low-engagement nurses were concentrated in resource-limited areas with weak team structures. These profiles emphasise the combined influence of leadership, relational support, and access to resources. They address Objectives 1, 4, and 5, by showing how these organisational elements intersect in ways that shape engagement.

Overall, the findings presented in Tables 3 and 4; Figs. 1, 2 and 3 provide a coherent picture of how organisational context shapes EBP across healthcare settings. Variation is shaped less by sector and more by the intersection of leadership, culture, social capital, and structural conditions.

## Discussion

This study examined how organisational context shapes the implementation of EBP across acute and primary care settings. Consistent with prior research, overall engagement with EBP was moderate and did not differ significantly between sectors, yet the distribution of contextual supports was uneven [8, 10]. Leadership behaviours were strongly associated with reported EBP use, and the mediation analysis indicates that this influence is partly channelled through social capital, where cohesive peer networks, mentoring, and shared goals support the translation of knowledge into everyday care [14, 15, 33]. These findings extend earlier English work by clarifying that leadership effects are not solely direct but operate through relational mechanisms that enable knowledge mobilisation. Earlier work in the English NHS has similarly shown that when managers actively champion research, team confidence in using evidence increases [12–14]. Our study expands this by demonstrating that the influence of leadership is not simply direct, but also operates through the development of social capital.

Social capital emerged as a key mediator in the relationship between leadership and evidence use. Teams with stronger peer networks, mentoring relationships, and shared goals reported higher EBP engagement. This reflects the growing body of literature recognising relational dynamics as central to knowledge mobilisation [14, 15]. Informal exchanges among colleagues often serve as practical vehicles for translating research into everyday care, particularly where formal structures are lacking [33]. Our findings reinforce this and highlight the need for organisational strategies that strengthen peer cohesion alongside formal leadership development.

Differences within acute care settings offer further nuance. Subgroup analysis revealed that units such as general medicine and ICU/CCU had higher scores across leadership, culture, and evaluation domains. These findings suggest that some specialties are better positioned structurally and relationally to support EBP. This supports previous observations that local context often determines whether system-wide policy goals are realised in practice [34, 35]. Emergency and surgical teams reported lower scores, reflecting known challenges around time pressure and fragmented workflows in those areas [36].

Despite national efforts to develop research-active environments through initiatives such as the NHS People Plan [17, 18], our findings suggest that such policy aims remain unevenly realised across clinical settings. Organisational culture, while not significantly different between sectors, appeared too weak in many areas to drive consistent behaviour change. Similarly, access to structural resources was limited, particularly where nurses lacked protected time, space, or technological support. These findings support calls for more investment in organisational infrastructure that enables not just encourages evidence use [2, 32].

Translating these findings into action requires a small set of feasible levers. First, formalise protected EBP and learning time within job plans and rota design, aligning with national workforce commitments in the NHS People Plan and NHS England’s research and evidence strategy [32]. Second, resource embedded facilitation within governance structures, for example ward or team based EBP facilitators and knowledge brokers, consistent with implementation guidance and systematic reviews showing that facilitation, local opinion leaders, and tailored support improve the uptake of evidence and guideline adherence [32, 37]. Third, invest in organisational knowledge infrastructure, including library and knowledge services and point-of-care evidence tools, which reviews identify as modifiable system resources that strengthen real-time access to evidence and evaluation mechanisms [32, 37]. Finally, expand clinical academic pathways for nurses and link progression to service improvement plans, as advocated in national strategies, to build the leadership and social capital highlighted by our mediation analysis [17, 18]. Overall, these steps operationalise the PARIHS emphasis on context and facilitation and target the leadership, relational, and infrastructure gaps observed in our data [30, 38].

Cluster analysis further confirmed that organisational factors do not operate in isolation. High EBP engagement was most common in environments that combined strong leadership, cohesive team relationships, and adequate resources. These findings align with earlier work by Estabrooks et al., [7] and Squires et al., [29], who argued that multiple contextual domains must converge to create implementation-ready environments. Similar conclusions have been drawn in recent English studies, where high-performing teams were found to rely on the interplay between managerial support, trust-based peer networks, and reliable infrastructure [8, 10, 26, 39]. This multi-dimensional insight offers a more complete picture of what implementation support requires at the frontline. Rather than focus solely on individual nurse capability, our findings argue for system-level interventions that strengthen relational, structural, and cultural foundations for evidence use.

### Limitations

This study draws on a large and diverse sample of nurses working in both acute and primary care, but several limitations must be acknowledged. The cross-sectional design captures organisational conditions and EBP behaviours at a single point in time [32]. While this approach offers a valuable snapshot, it does not allow for causal conclusions or exploration of change over time [34]. A longitudinal design could better clarify how organisational changes influence evidence engagement. The use of self-reported data may introduce bias, as participants might overstate their engagement with EBP or organisational support [31]. Although validated instruments were used, social desirability remains a potential concern. Recruitment relied on voluntary participation, which may have resulted in a sample more interested in research or organisational improvement than the wider nursing population. The study excluded mental health services, meaning that findings cannot be generalised beyond generalist acute and primary care. Finally, while the ACT and EBPIS are widely used, their adaptations for the English healthcare system, while carefully implemented, may still carry measurement limitations. Despite these issues, the study offers important insight into the contextual factors shaping EBP implementation in nursing.

### Implications for practice and policy

Strengthening EBP hinges on shaping organisational context rather than adding more individual training. Within the English NHS, four complementary actions are practicable and mutually reinforcing. Organisations should incorporate protected EBP and learning time into job plans and rota templates and oversee delivery through routine governance, so time is realised in practice, consistent with national workforce commitments and evidence that infrastructure enables uptake. They should commission internal facilitation capacity with a defined remit in clinical governance, for example team based EBP facilitators or advanced practice leads, reflecting guidance and reviews that show facilitation and local opinion leadership support implementation and culture change. Knowledge infrastructure should be treated as core capability, with funded library and knowledge services and point-of-care evidence tools to strengthen real-time access to research and evaluation mechanisms. Finally, clinical academic pathways for nurses should be expanded and explicitly tied to service improvement objectives to build the leadership and social capital identified in our mediation analysis. At system level, commissioners and integrated care systems can promote equity and accountability by ensuring consistent access to these resources across acute providers and primary care networks and by tracking EBP readiness with unit or PCN level indicators based on ACT domains and EBPIS use.

### Recommendations for future research

Longitudinal studies are needed to test how changes in leadership, social capital, and infrastructure affect sustained EBP engagement and to strengthen causal inference. Qualitative work could illuminate how nurses experience facilitation, protected time, and knowledge services in practice and how these interact with team dynamics. Further research should unpack the specific mechanisms by which social capital enhances evidence use across settings and roles, and evaluate context-responsive interventions that integrate facilitation, protected time, and knowledge infrastructure at unit or network level.

### Conclusion

Organisational context is central to how nurses engage with EBP. While average engagement was comparable across sectors, leadership, relational cohesion, and access to structural resources varied and were decisive. Social capital mediated the impact of leadership, and differences across acute specialties emphasised the importance of local conditions. Future efforts should prioritise creating enabling environments in which evidence use is structurally supported, relationally reinforced, and routinely expected.

## Data Availability

The datasets utilised and/or analysed in this study are accessible from the corresponding author upon reasonable request. All data included in this document have not been previously published.

## Abbreviations

EBP: Evidence-Based Practice
EBPIS: Evidence-Based Practice Implementation Scale
ACT: Alberta Context Tool
NHS: National Health Service
NMC: Nursing and Midwifery Council
NICE: National Institute for Health and Care Excellence
ICU: Intensive Care Unit
CCU: Coronary Care Unit
SD: Standard Deviation
CI: Confidence Interval
ANOVA: Analysis of Variance
RMSEA: Root Mean Square Error of Approximation
CFI: Comparative Fit Index
PCN: Primary Care Network
PARIHS: Promoting Action on Research Implementation in Health Services
